# An absence of platelet activation following thalidomide treatment *in vitro* or *in vivo*

**DOI:** 10.18632/oncotarget.16205

**Published:** 2017-03-15

**Authors:** Jianlin Qiao, Yulu Wu, Xiaoqing Wu, Yun Liu, Xiaoqian Li, Wen Ju, Kunming Qi, Depeng Li, Elizabeth E. Gardiner, Robert K. Andrews, Lingyu Zeng, Kailin Xu

**Affiliations:** ^1^ Blood Diseases Institute, Xuzhou Medical University, Xuzhou, China; ^2^ Department of Hematology, The Affiliated Hospital of Xuzhou Medical University, Xuzhou, China; ^3^ Key Laboratory of Bone Marrow Stem Cell, Jiangsu Province, Xuzhou, China; ^4^ Department of Cancer Biology and Therapeutics, John Curtin School of Medical Research, Australian National University, Canberra, Australia; ^5^ Australian Centre for Blood Diseases, Monash University, Melbourne, Australia

**Keywords:** thalidomide, platelet activation, blood coagulation, thrombosis, multiple myeloma

## Abstract

Increased risk of thromboembolism and platelet hyperreactivity has been reported in patients receiving thalidomide therapy. Whether thalidomide induces platelet activation directly or through other factors remains unclear. The aim of this study was to evaluate the effect of thalidomide on platelet activation under resting conditions *in vitro* and *in vivo*. Isolated human or mouse platelets were treated with different concentrations of thalidomide (10, 50 and 100 μg/ml) for 60 min at 37°C followed by analysis of platelet surface expression of platelet receptors GPIbα, GPVI, α_IIb_β_3_ and P-selectin, and PAC-1 or fibrinogen binding, by flow cytometry and collagen- or ADP-induced platelet aggregation. In addition, thalidomide (200 mg/kg) was intraperitoneally injected into mice for analysis of the effect of thalidomide on platelet activation *in vivo*. No increased expression of P-selectin, PAC-1 or fibrinogen binding was observed in either human and mouse platelets after thalidomide treatment *in vitro* for 60 min at 37°C. Thalidomide treatment also did not affect expression of GPIbα, GPVI or α_IIb_β_3_, nor did it affect collagen- or ADP-induced platelet aggregation at threshold concentrations. However, while mice injected with thalidomide displayed no increased surface expression of platelet P-selectin or α_IIb_β_3_, there was a significantly shortened tail bleeding time, thrombin time, prothrombin time together with higher levels of Factor IX and fibrinogen. In conclusion, thalidomide at therapeutic doses does not directly induce platelet activation under resting conditions *in vitro* or *in vivo*, but results in increased procoagulant activity, which could explain the thalidomide-dependent prothrombotic tendency in patients.

## INTRODUCTION

As a synthetic glutamic acid derivative, thalidomide was originally prescribed as a sedative and antinausea medicine in pregnant women, but was withdrawn from the market as severe birth defects were observed in patients receiving thalidomide [1, 2]. However, thalidomide has returned to clinical use in recent years for the treatment of several proinflammatory or autoimmune diseases since it was discovered to modulate immune and inflammatory responses [3, 4]. In addition, thalidomide has been demonstrated to be effective in the treatment of multiple myeloma (MM) presumably due to antiangiogenic activity or immune modulation [5]. However, its efficacy is largely limited by the increased incidence of thrombosis, especially when used in combination with other drugs, such as dexamethasone or doxorubicin [6, 7]. Meanwhile, addition of thalidomide has been demonstrated to increase the incidence of 2-year thrombosis from 1.5% to 18.5% in patients administered melphalan or prednisone [8].

Given the association of thalidomide with increased thrombotic risk [9], the precise mechanism by which thalidomide induces thrombogenesis *in vivo* remains poorly understood. Thrombotic manifestations observed in patients treated with thalidomide are usually venous; however arterial events have also been reported in some individuals [10]. Under physiological conditions, the vascular endothelium mediates vascular dilatation, prevents platelet adhesion and activation, blocks thrombin formation and mitigates fibrin deposition to maintain an anti-thrombotic state [11]. When the endothelium is activated or injured, disrupting these processes, this may lead to a prothrombotic state. A previous study reported that increased endothelial activation was observed in MM patients treated with thalidomide as demonstrated by elevated levels of von Willebrand factor [12], a surrogate marker of endothelial cell injury [13]. Furthermore, increased expression of phosphatidylserine, tissue factor and enhanced thrombin generation were observed in cultured human umbilical vein endothelial cells treated with thalidomide and plasma from MM patients [14], suggesting thalidomide potentially induces thrombogenesis by disrupting the balance between pro-coagulant and anticoagulant proteins on the surface of endothelial cells. Apart from the endothelium, aberrant activation of platelets was also reported in MM patients receiving thalidomide as shown by increased platelet surface P-selectin expression [15], PAC-1 binding [16] and platelet aggregation [17]. However, the exact role of thalidomide on platelet activation has not been thoroughly investigated, and whether thalidomide induces platelet activation directly or through other factors remains poorly understood. In this study, we investigate the potential role of thalidomide on platelet activation *in vitro* and *in vivo*, by directly treating isolated human or mouse platelets with thalidomide, and by analyzing platelets and coagulation markers following injection of thalidomide into mice. Together, our findings support a mechanism involving a predominantly procoagulant effect rather than a direct effect on platelets, with implications for the future clinical use of thalidomide.

## RESULTS AND DISCUSSION

**Figure 1:**
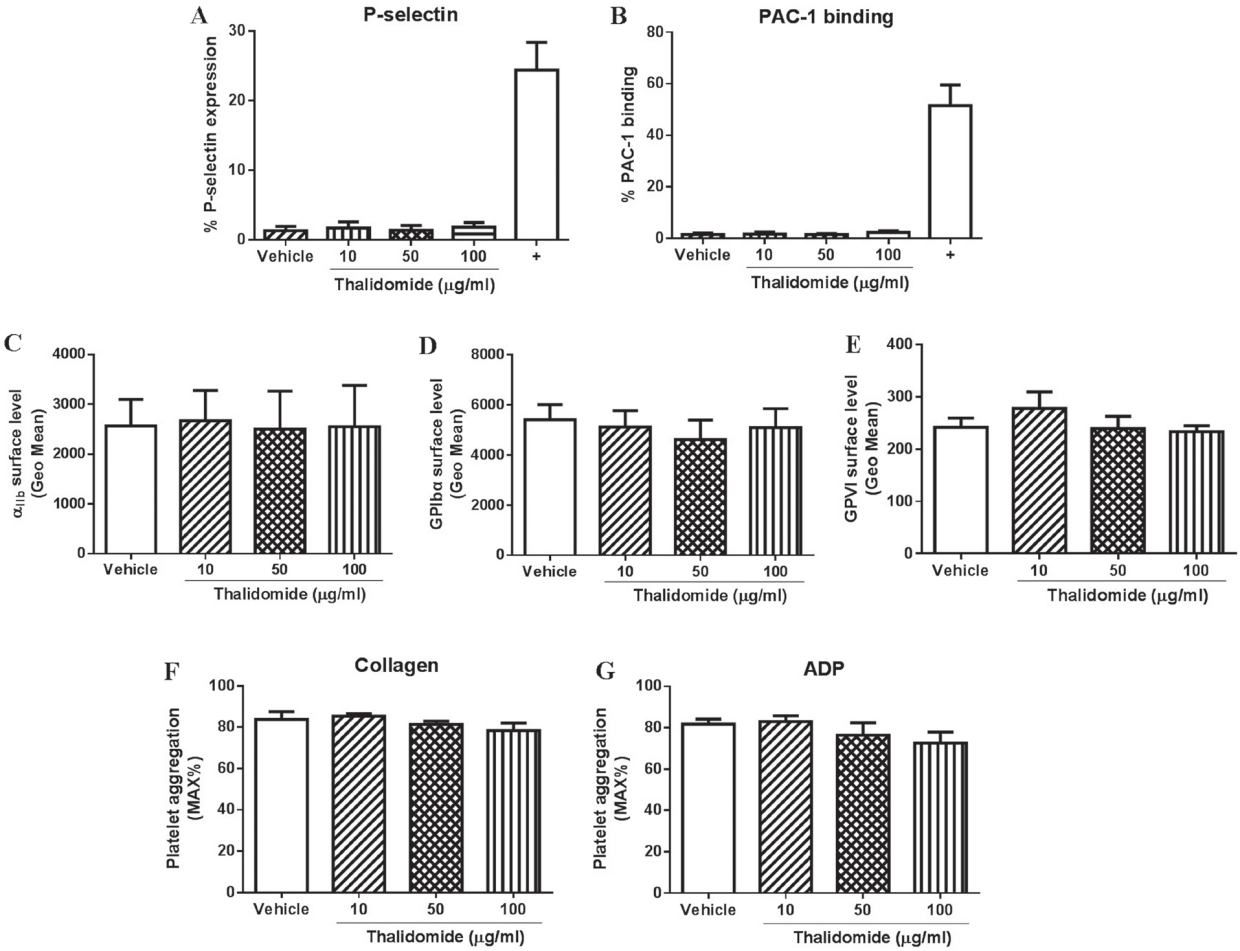
The effect of thalidomide on human platelets *in vitro* Isolated human platelets (2.5 × 10^8^/ml) were treated with 10, 50 or 100 μg/ml thalidomide for 1 h at 37°C followed by measuring (**A**) expression of P-selectin, (**B**) PAC-1 binding, or expression of **(C**) α_IIb_, (**D**) GPIbα or (**E**) GPVI by flow cytometry. In addition, platelet aggregation in citrated PRP after thalidomide treatment in response to threshold concentration of (**F**) collagen (2.5 μg/ml) or (**G**) ADP (5 μM) was also performed. Collagen (10 μg/ml) was used as a positive control (“+”) for platelet activation. All the data were acquired from at least three independent experiments using different donors.

Several studies have demonstrated increased platelet activation in patients receiving thalidomide therapy [15, 16]. To investigate whether thalidomide was directly activating platelets, we treated washed platelets from healthy individuals with different concentrations of thalidomide (10, 50 and 100 μg/ml) *in vitro* and evaluated the extent of platelet activation. As shown in Figure 1A, there was no platelet activation observed following thalidomide treatment as compared with vehicle controls, demonstrated by no significant increase of expression of P-selectin on the platelet surface. In addition, platelet integrin α_IIb_β_3_ activation (essential for platelet aggregation) was also measured and showed no increased PAC-1 binding to platelets treated with thalidomide (Figure 1B), indicating thalidomide did not lead to activation of platelet α_IIb_β_3_ *in vitro*.

Platelet receptors, such as GPIbα, GPVI or integrin α_IIb_β_3_ play an important role in regulating platelet function and aberrant expression of these receptors has been shown to affect platelet thrombus formation [18, 19]. As seen in Figure 1C–1E, there was no significant changes in expression of α_IIb_, GPIbα or GPVI on platelets treated with thalidomide compared with vehicle controls when measured by flow cytometry, revealing thalidomide also does not alter the expression profiles of these important platelet receptors.

**Figure 2:**
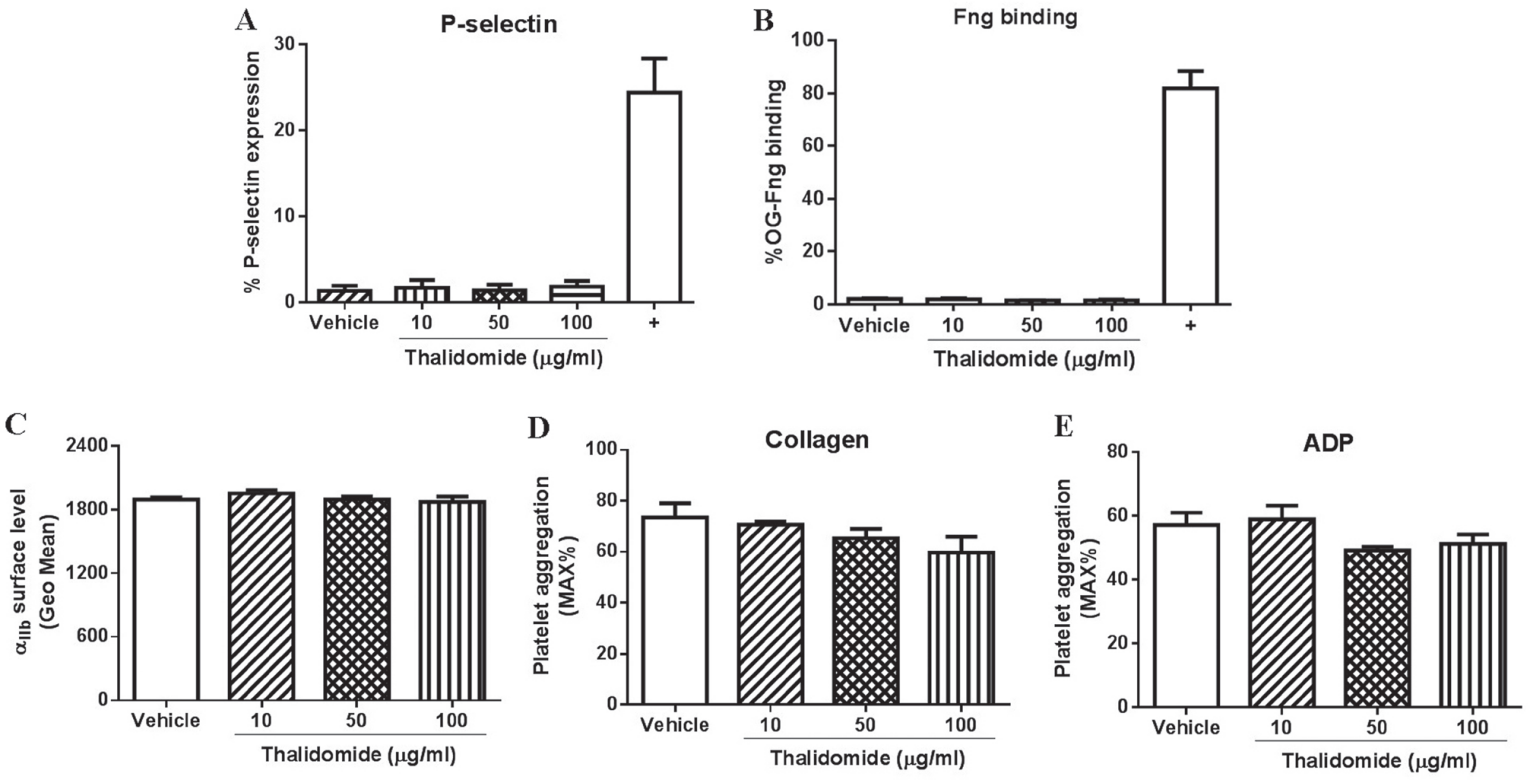
The effect of thalidomide on mouse platelets *in vitro* Isolated mouse platelets (2.5 × 10^8^/ml) were treated with indicated concentrations of thalidomide followed by measuring (**A**) expression of P-selectin, (**B**) fibrinogen (Fng) binding, or (**C**) expression of α_IIb_ by flow cytometry. Platelet aggregation in mouse washed platelets after thalidomide treatment in response to threshold concentration of (**D**) collagen (1 μg/ml) or (**E**) ADP (10 μM) was performed in the presence of fibrinogen (0.5 mg/ml). CRP (10 μg/ml) was used as a positive control (“+”) for mouse platelet activation. All the data were acquired from at least three independent experiments.

Consistent with the lack of effect of thalidomide on platelet activation or receptor expression, pre-treatment of platelets with different concentrations of thalidomide also showed no effect on collagen- or ADP-induced platelet aggregation at threshold concentrations in human citrated PRP (Figure 1F–1G). Further, consistent with these findings in human platelets, thalidomide-treated mouse platelets also displayed no sign of activation as evaluated by P-selectin expression or fibrinogen binding (Figure 2A–2B), while α_IIb_ surface expression and collagen- or ADP-induced platelet aggregation at threshold concentrations were similarly unaffected (Figure 2C–2E). Taken together, these data demonstrate that thalidomide does not induce platelet activation *in vitro* under experimental conditions described.

**Figure 3:**
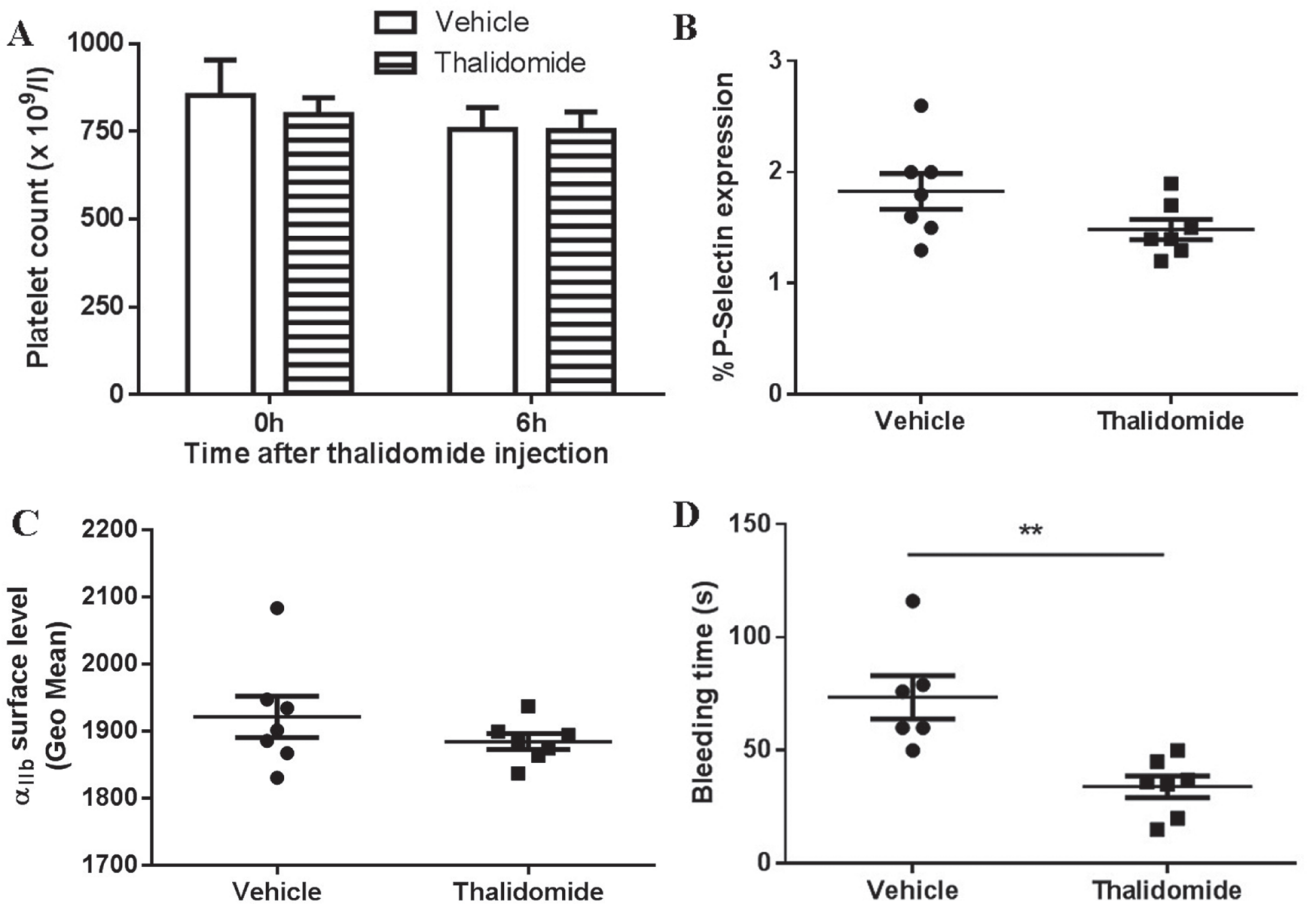
The effect of thalidomide on platelets *in vivo* Thalidomide (200 mg/kg) was intraperitoneally injected into mice followed by collection of peripheral blood at 6 h after administration for analysis of (**A**) platelet count, (**B**) platelet surface P-selectin expression and (**C**) α_IIb_ expression. (**D**) Tail bleeding time was also measured. **P < 0.01 (Unpaired student *t-test*). All the data were acquired from at least three independent experiments.

**Figure 4:**
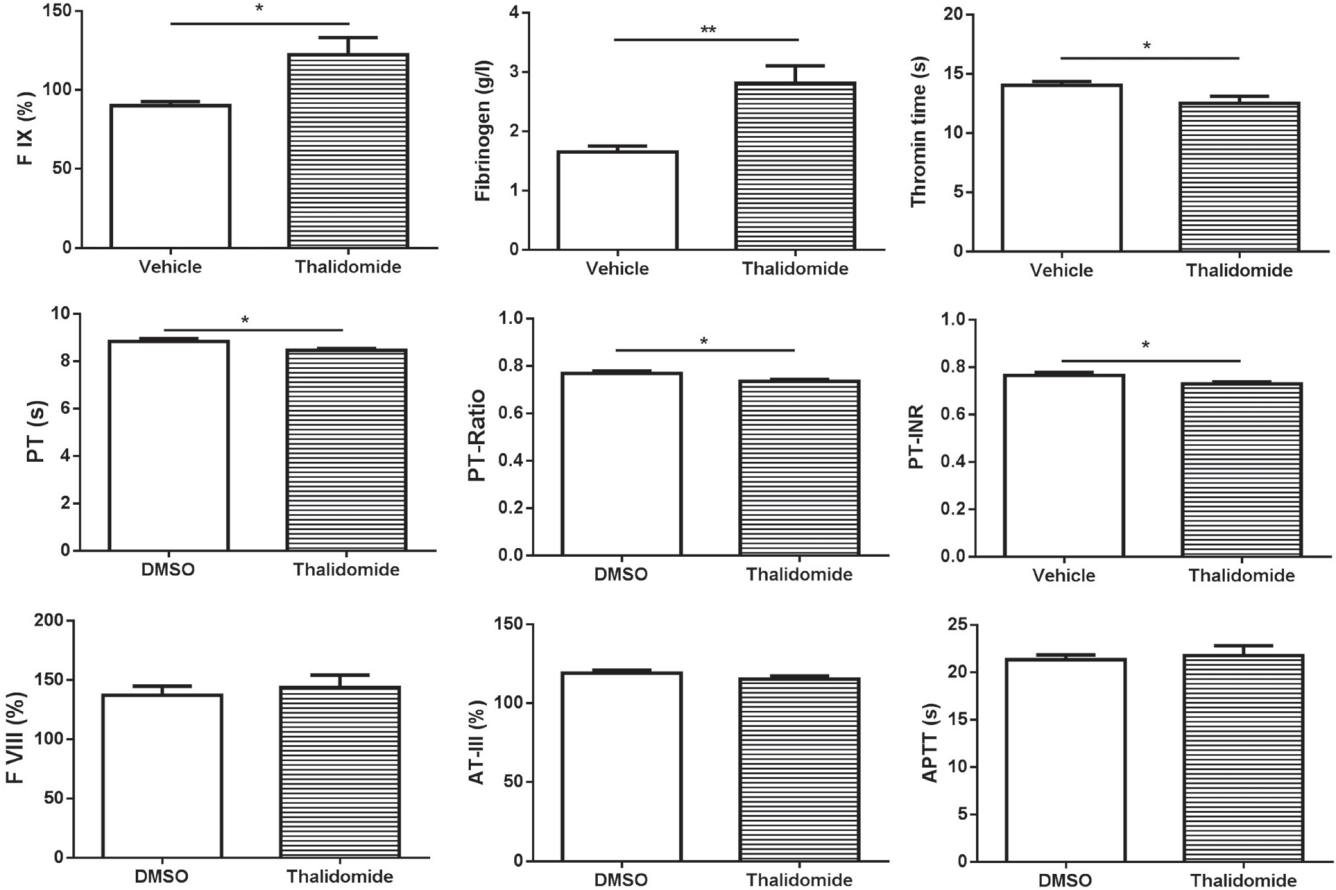
Analysis of coagulation parameters following thalidomide administration into mice Plasma was isolated from mice after thalidomide administration for 6 h followed by analysis of the level of factor IX (FIX), fibrinogen, thrombin time (TT), prothrombin time (PT), PT-Ratio, PT-INR, FVIII, AT-III, and APTT using an automated coagulation analyzer. *P < 0.05 (Unpaired student *t-test*). **P < 0.01 (Unpaired student *t-test*). PT-INR: Prothrombin Time-International Normalized Ratio; APTT: Activated Partial Thrombin Time; AT: Anti-thrombin.

In order to investigate whether thalidomide affects platelet activation *in vivo*, thalidomide was intraperitoneally injected into mice followed by collection of peripheral blood at 6 h after thalidomide administration. We found no significant changes of platelet count after thalidomide injection (Figure 3A), and no platelet activation or changes of α_IIb_ surface levels (Figure 3B–3C), consistent with the *in vitro* findings. Interestingly, however, mice injected with thalidomide displayed a significantly shortened tail bleeding time compared to mice receiving vehicle alone (Figure 3D), suggesting thalidomide might affect coagulation *in vivo*. To further investigate this possibility, plasma was isolated from mice injected with thalidomide or vehicle and coagulation-related parameters were analysed. As shown in Figure 4, significantly increased levels of factor IX (FIX) (122.30 ± 10.76%) *(P* < 0.05) and fibrinogen (2.81 ± 0.30 g/l) (*P* < 0.01) were observed in mice after thalidomide administration compared with vehicle (90.06 ± 2.46% for FIX and 1.65 ± 0.10 g/l for fibrinogen). In addition, thrombin time (TT) (12.50 ± 0.60 s) (*P* < 0.05), prothrombin time (PT) (8.46 ± 0.09) (*P* < 0.05), PT-Ratio (0.74 ± 0.01) (*P* < 0.05) and normalized prothrombin time (PT-INR) (0.73 ± 0.01) (*P* < 0.05) of mice injected with thalidomide were significantly shortened compared with mice receiving vehicle (14.03 ± 0.32 s for TT, 8.83 ± 0.12 for PT, 0.77 ± 0.01 for PT-Ratio, and 0.77 ± 0.01 for PT-INR). Interestingly, thalidomide treatment did not alter the levels of coagulation factor VIII (FVIII), anti-thrombin III (ATIII) as well as the activated partial thrombin time (APTT) (Figure 4). Taken together, our study demonstrates that thalidomide induces increased pro-coagulant activity in mice, which was consistent with a previous study demonstrating increased expression of tissue factor and phosphatidylserine exposure on the surface of endothelial cells and monocytes by a thalidomide-based regimen [20].

In conclusion, this study demonstrates that thalidomide dose not trigger platelet activation *in vitro* or *in vivo*. An important limitation of our study is that it used platelet samples from healthy humans and wildtype mice not afflicted with disease. Thus, increased platelet activation in patients receiving thalidomide therapy might be due to the underlying disease, for example, making platelets more susceptible to activation, or an indirect effect of coagulopathy. Further studies using platelets isolated from patients with active myeloma disease are required to confirm that thalidomide treatment does not directly affect platelet function. A further possibility not excluded by our results is that thalidomide treatment indirectly leads to platelet hyperreactivity by a mechanism involving activation of endothelial cells. However, further studies are required to confirm these hypotheses.

Whilst the thalidomide/thrombosis mechanistic link has not been fully elucidated here, the data obtained in our study highlight that thalidomide does not trigger or enhance platelet reactivity. That is, our data indicate that the effect is not via direct influence on platelet reactivity or disruption of normal platelet responsiveness. Further, our data suggest that thalidomide treatment is able to modulate the procoagulant state. This is potentially significant for the clinical use of thalidomide as an anti-inflammatory therapeutic and in the setting of neoplasia, particularly when anti-coagulant/antiplatelet therapies form part of the clinical management of patients with complex disease aetiologies. Our data, for example, may be relevant for management of prophylactic anticoagulation in patients with myeloma receiving thalidomide as part of their treatment regime.

## MATERIALS AND METHODS

### Reagents

Thalidomide was purchased from Changzhou Pharmaceutical Factory Co., Ltd (Changzhou, Jiangsu, China). Collagen and ADP were from Helena laboratories (Beaumont, Texas, USA). Collagen-related peptide (CRP) was prepared as previously described [21]. FITC-conjugated anti-human CD41a and PAC-1 antibody were from BD Biosciences (San Jose, CA) and Becton Dickinson (San Jose, CA) respectively. PE-conjugated anti-human/mouse CD62p (P-Selectin) and purified anti-human Glycoprotein (GP) VI antibody were purchased from eBioscience (San Diego, CA). FITC-conjugated anti-CD42b antibody was from Abcam (Cambridge, MA). FITC-conjugated goat anti-mouse IgG was from ZSGB-BIO (Beijing, China). Oregon Green-labelled fibrinogen (OG-Fng) was from Thermo Fisher Scientific (Waltham, MA).

### Platelet preparation

All procedures involving collection of human and mouse blood were approved by the Medical Ethics Committee of Xuzhou Medical University. Informed consent has been obtained and all clinical investigations have been conducted in accordance with the ethical standards and according to the principles expressed in the Declaration of Helsinki. Human or mouse platelets were isolated as previously described [22, 23]. Briefly, venous human blood was collected into trisodium citrate and centrifuged to obtain platelet-rich plasma (PRP). For washed platelet studies, blood was collected into acid-citrate-dextrose (ACD) and centrifuged to get PRP. Platelet were washed three times in citrate-glucose-saline (CGS) and resuspended in Tyrode's buffer. Mouse platelets were isolated from ACD anti-coagulated blood, washed using CGS buffer and resuspended in Tyrode's buffer.

### Platelet activation

Platelet activation was evaluated by measuring the surface expression of the α-granule glycoprotein, P-selectin, and by the activation-dependent binding of either PAC-1 (human) or fibrinogen (mouse) to platelet α_IIb_β_3_. Isolated human or mouse platelets (2.5 × 10^8^/ml) were incubated with different concentrations of thalidomide (10, 50 or 100 μg/ml) according to published studies [24, 25] for 60 min at 37°C followed by addition of PE-conjugated anti-P-selectin antibody (2.5 μg/ml), FITC-conjugated PAC-1 antibody (1 μg/ml), or Oregon Green-labelled fibrinogen and analysed by flow cytometry. Collagen (10 μg/ml) and CRP (10 μg/ml) were used as a positive control for human and mouse platelet activation, respectively.

### Surface expression of platelet receptors

Washed platelets were untreated or treated with thalidomide for 60 min at 37°C, then FITC-conjugated anti-CD42b antibody (GPIbα) (1 μg/ml), FITC-conjugated mouse anti-human CD41a antibody (α_IIb_) (10 μl/test) or anti-human GPVI antibody (2.5 μg/ml) (detected by FITC-conjugated goat anti-mouse IgG) were added and incubated for 30 min at room temperature and analysed by flow cytometry. FITC-conjugated anti-mouse CD41a antibody (2 μg/ml) was used for analysing α_IIb_ surface expression on mouse platelets.

### Platelet aggregation

Platelet aggregation was performed using human citrated PRP or mouse washed platelets. After thalidomide treatment as described above, human or mouse platelet aggregation (250 × 10^6^/ml) in response to collagen or adenosine diphosphate (ADP) at threshold concentrations (defined as the lowest dose that induced more that 50% of aggregation) was determined by light transmittance aggregometry (Helena Aggram, Helena Laboratories, Beaumont, USA) as previously described [22]. The extent of platelet aggregation was defined as the percentage of maximum platelet aggregation (monitored for 5 min) determined in the absence of drug. Mouse washed platelet aggregation was performed in the presence of fibrinogen (0.5 mg/ml).

### *In vivo* studies in mice

BALB/c mice, aged 10–12 weeks and weighted 20–25 g, were purchased from SLAC Laboratory Animal, Shanghai, China. The mice were housed in sterilized cages at the Experimental Animal Center of Xuzhou Medical University. The experiments on animals were performed in accordance with the Xuzhou Medical University concerned with the ethics of experimentation. Thalidomide (200 mg/kg) was intraperitoneally injected into mice followed by collecting peripheral blood after 6 h for analysis of platelet count by an automatic blood cell analyzer (BC5300, Mindray, Shenzhen, Guangdong, China), and platelet surface expression of P-selectin, platelet receptor α_IIb_ expression by flow cytometry as described above. An equal volume of dimethyl sulfoxide (DMSO) was used as vehicle only control.

### Tail bleeding time

At 6 h following thalidomide or DMSO administration, a 10-mm segment of tail tip of each mouse was cut off and the tail was immersed in pre-warmed sterile saline solution (37°C). Tail bleeding time was defined as the time taken for bleeding to stop.

### Coagulation analysis

Plasma was isolated from mice treated with thalidomide or DMSO for analysis of the level of factor IX factor VIII, anti-thrombin III, and fibrinogen, and for determining the thrombin time, activated partial thrombin time and prothrombin time using an automated coagulation analyzer (Sysmex CS-5100).

### Statistical analysis

Data were represented as mean ± standard error (SE). At least three independent experiments were performed for each assay. For comparison between vehicle and different concentrations of thalidomide, data were assessed by one-way ANOVA with Tukey multiple comparison post-hoc analysis using GraphPad Prism software. Two tailed student *t-test* was used to evaluate the difference between vehicle and thalidomide-injected mouse. *P* < 0.05 indicates statistically significance.

## Abbreviations

MM: multiple myeloma
CRP: collagen-related peptide
ADP: adenosine diphosphate
OG-Fng: Oregon Green-labelled fibrinogen
PRP: platelet-rich plasma
